# Impact of Longitudinal Pulmonary Ultrasound Curriculum in a Multi-disciplinary Critical Care Fellowship

**DOI:** 10.7759/cureus.89447

**Published:** 2025-08-05

**Authors:** Sam Chiacchia, Melissa D'souza, Jai Madhok, Natalie Htet

**Affiliations:** 1 Emergency Medicine, Stanford University School of Medicine, Stanford, USA; 2 Critical Care Medicine, Stanford University School of Medicine, Stanford, USA; 3 Critical Care Medicine, Anesthesiology, Perioperative and Pain Medicine, Stanford University School of Medicine, Stanford, USA; 4 Emergency Medicine and Critical Care Medicine, Stanford University School of Medicine, Stanford, USA

**Keywords:** competency based medical education, pocus (point of care ultrasound), pulmonary critical care, respiratory disease, resuscitation

## Abstract

Point-of-care ultrasound (POCUS) has become a central component in the assessment and management of critically ill patients. Despite its widespread application, there is no standardized curriculum across critical care fellowships. Previous studies have examined the efficacy of ultrasound curricula in enhancing provider comfort and expertise with POCUS. However, these educational interventions are typically limited to specific critical care subspecialties and do not evaluate the longitudinal impact of POCUS training.

In this study, we assessed the impact of a longitudinal pulmonary ultrasound curriculum on a multidisciplinary group of critical care fellows. Participants trained in internal medicine, emergency medicine, anesthesia, and neurology first completed a pre-training knowledge assessment. They then attended two one-hour didactic lectures on lung ultrasound (LUS). Hands-on training sessions were provided, with each fellow receiving two-hour scanning sessions under the guidance of critical care ultrasound faculty.

Confidence among critical care fellows in acquiring and interpreting images increased from 57% (n = 21) to 79% (n = 19). Similar increases in fellow confidence were noted in using LUS to identify the etiology of respiratory distress and using ultrasound for the diagnostic and therapeutic management of pleural effusions. All study participants were able to accurately complete a comprehensive LUS exam within 10 minutes after training. Quality improvement initiatives and scan reviews provided ongoing feedback over the next year. Upon follow-up with graduates within a year of completing their fellowship, pulmonary ultrasound was routinely used in their practice, with an average frequency of two to three times per week. All respondents reported that pulmonary ultrasound training during fellowship had meaningfully changed how they integrate the tool into their clinical practice.

## Introduction

Point-of-care ultrasound (POCUS) has become increasingly important for clinicians due to its reproducibility in diagnosing life-threatening conditions, as well as its speed, non-invasiveness, and relative affordability [1-4]. Lung ultrasound (LUS) is an important assessment of critically ill patients, frequently modifying provider differentials and guiding patient care [5-8]. Integrating LUS with cardiac ultrasound is highly valuable for diagnosing patients with respiratory distress and assessing conditions such as pneumothorax, pulmonary edema, pleural effusion, and pneumonia [9]. Despite its common application and recommended use by the Accreditation Council for Graduate Medical Education (ACGME), there is no standardized curriculum among critical care fellowships [10].

Previous studies have explored the impact of ultrasound curricula in increasing trainee comfort and expertise with POCUS [7]. Killu et al. demonstrate that, within a single pulmonary critical care training program, implementation of POCUS training modified diagnoses approximately two-thirds of the time and changed management approximately one-third of the time among patients studied [6]. Similarly, Jalivand et al. report that a short-term training module improved provider expertise with POCUS in a group of surgical critical care residents [11]. These studies establish important context and demonstrate that a structured approach to POCUS training can yield meaningful returns for trainees. However, their evaluation of POCUS training modules within specific critical care subspecialties, such as surgical or pulmonary critical care, may limit the generalizability of their results. Perhaps more importantly, as critical care becomes increasingly multidisciplinary, there is a growing need for a POCUS curriculum well suited for training providers from diverse training backgrounds.

There are a few studies that assess the impact of POCUS education on critical care trainees, and even less literature that evaluates the longitudinal impact of this education. In their meta-analysis of over 5000 published abstracts, Rajamani et al. reveal that less than 1% of studies assessed the longitudinal impact of POCUS training [12]. Thus, there is motivation among POCUS educators, particularly in critical care, to explore the impact of longitudinal educational interventions. Here, we chose to focus on advanced pulmonary ultrasound, specifically the integration of pulmonary ultrasound with cardiac ultrasound, given its importance in managing critically ill patients in the ICU. In this study, we aimed to address two limitations in the current literature by evaluating the impact of a longitudinal POCUS training model among a group of multidisciplinary critical care fellows. Our primary outcome of interest was trainee confidence with the application of ultrasound in the diagnosis and management of pulmonary pathology in critically ill patients. Our secondary outcomes included time to acquisition of comprehensive LUS images. 

Of note, the rationale and results from this study were presented as an oral presentation at the 6th Annual Medical and Bioscience Education Day/Stanford Institute for Medical & Bioscience Education Conference (SIMEC) VIII (Stanford, CA, USA) on May 13, 2023.

## Materials and methods

This prospective study was conducted from July 1, 2022, to July 1, 2024, at Stanford Hospital Center (SHC), a quaternary academic hospital in Palo Alto, California. Institutional Review Board (IRB) exemption was obtained (see Appendix A). The study’s inclusion criteria were enrollment in Stanford School of Medicine’s multidisciplinary critical care medicine fellowship class of 2022-2023; there were no participants excluded from the study based on exploratory aims of the project and small sample size. The resulting study cohort included 21 subjects from the class of 2022-2023. All fellows received a year-long critical care ultrasound curriculum covering advanced cardiac, lung, intracranial, and abdominal examinations. Hands-on scanning of patients with relevant pathology with the critical care ultrasound faculty was provided, with each fellow receiving a minimum of two hours of scanning training. Quality review, led by trained ultrasound faculty in the critical care ultrasound fellowship program, provided structured review and longitudinal feedback for trainees over the year. Quizzes and questionnaires with ultrasound images and clinical vignettes were sent every other week to reinforce learning.

A 28-question pre-training survey was administered to fellows to assess demographics, confidence, and knowledge of POCUS LUS (see Appendix B). A post-training test with a similar structure was conducted to evaluate changes in knowledge and confidence. The questions were designed based on LUS learning objectives and the bedside lung ultrasound in emergency (BLUE) protocol [13-17]. Six questions focused on demographics and needs assessment, while the confidence assessment questionnaire measured self-perceived competence. Knowledge-based questions covered knobology, normal lung anatomy, and pathological findings such as pneumothorax, pulmonary edema, pneumonia, pleural effusion, and diaphragm excursion, using both images and videos. Content validity of the survey was reviewed by three members of critical care ultrasound experts (one each from cardiology, critical care medicine, anesthesia critical care medicine, and emergency medicine critical care).

After completing the survey, participants attended two one-hour didactic LUS lectures delivered by an ultrasound faculty member trained in emergency medicine and critical care. During the didactic LUS tutorial, fundamental principles and operational techniques for LUS examinations were explained. The session also covered pathologies such as diaphragm weakness, endotracheal tube confirmation, pneumothorax, pleural effusion, interstitial syndrome within the BLUE protocol, integration with cardiac ultrasound, and deep vein thrombosis (DVT) [13-17].

**Figure 1:**
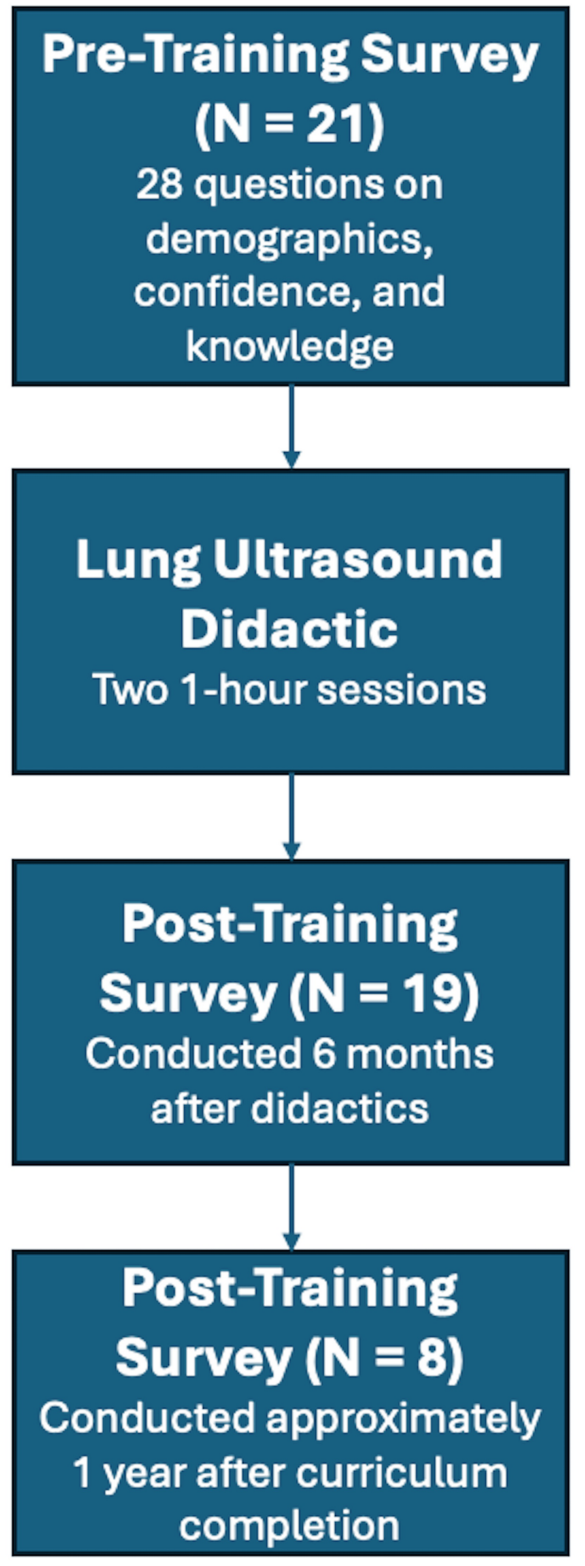
Study timeline

A post-survey was sent out to fellows six months after the pre-survey. A final survey was developed and sent to graduates on July 1, 2024 (one year after completion of the curriculum) to assess their sentiments regarding the impact of the pulmonary ultrasound curriculum on their practice patterns as critical care attendings. The questions were rated on a 5-point Likert scale, with the possible answers being strongly disagree (1 point), disagree (2 points), neutral (3 points), agree (4 points), and strongly agree (5 points). Our study timeline is summarized in Figure 1. 

Analysis of the resulting data was completed using Microsoft Excel (Microsoft Corp., Redmond, WA, USA). Given the descriptive aims of this pilot study, we did not conduct formal hypothesis testing to assess the statistical significance of the educational intervention. Participant age and gender were not recorded, as they were not expected to offer meaningful or actionable insights into the study outcomes. Instead, cohort demographic data, including previous residency training and postgraduate training level, were tabulated to provide insight into cohort generalizability and potential confounders, such as previous exposure and training in POCUS.

## Results

The pre-training survey was completed by 21 fellows, and the post-training survey was completed by 19 fellows. The study had more first-year participants in the pre- and post-surveys, with 85% (n = 18) and 84% (n = 16) of pre- and post-training surveys completed by first-year fellows, respectively. Emergency medicine-trained critical care fellows made up 43% (n = 9) of respondents, internal medicine-trained fellows made up 38% (n = 8), and neurology-trained fellows and anesthesia-trained fellows were 10% (n = 2), respectively (Table 1). Most participants were in postgraduate year (PGY) five. Around 52% of survey takers reported prior training in thoracic ultrasound (n = 11). All fellows agreed that pulmonary ultrasound is an important part of critical care training before completing the training modules (n = 21).

**Table 1:** Participant training and clinical backgrounds PGY: Postgraduate year

Prior residency training	Pre-training (n = 21)	Post-training (n = 19)	Median PGY
Emergency medicine	9 (43%)	4 (21%)	5
Internal medicine	8 (38%)	9 (47%)	5
Anesthesia	2 (9.5%)	5 (26%)	5
Neurology	2 (9.5%)	1 (5.3%)	6.5

After the lecture and hands-on training, the time required to perform LUS decreased, with 100% (n = 19) of respondents able to complete the exam within five to 10 minutes (Table 2). Confidence in thoracic ultrasound image acquisition improved from 57% (n = 12) to 79% (n = 15) after training in integrating cardiac ultrasound with LUS (Table 3). Confidence in managing critically ill patients with respiratory distress using integrated cardiac and pulmonary ultrasound improved from 47% (n = 10) to 63% (n = 12) (Table 3). Confidence in evaluating pleural effusions increased from 66% (n = 14) to 100% (n = 19). Confidence in evaluating extubation readiness with lung and diaphragm ultrasound improved from 9.5% (n = 2) to 21% (n = 4). There was no change in confidence with respect to endotracheal tube (ETT) placement.

**Table 2:** Time to perform comprehensive LUS pre- and post-training LUS: Lung ultrasound

Time to LUS completion	Pre-training (n = 21)	Post-training (n = 19)
Five to 10 minutes	15 (71%)	19 (100%)
Unknown time	6 (29%)	0 (0%)

**Table 3:** Participant self confidence in using LUS LUS: Lung ultrasound, POCUS: Point-of-care ultrasound, ETT: Endotracheal tube

Parameters	Pre-training (n = 21)	Post-training (n = 19)
LUS image acquisition	12 (57%)	15 (79%)
Integration of LUS with cardiac POCUS in the management of respiratory distress	10 (47%)	12 (63%)
POCUS for confirmation of ETT placement	3 (14%)	3 (16%)
LUS for diagnostic/therapeutic management of pleural effusion	14 (66%)	19 (100%)
LUS for assessment of extubation readiness	2 (10%)	4 (21%)

**Figure 2:**
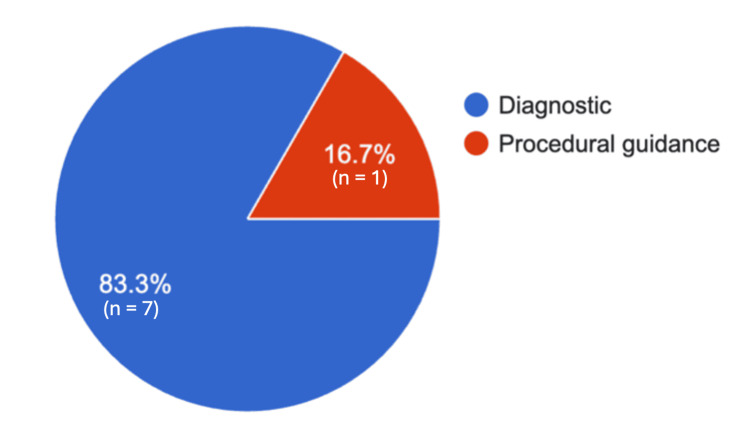
Graph displaying LUS application one year after fellowship training LUS: Lung ultrasound

In the final survey sent one year after critical care ultrasound training, eight responses were obtained. Lung ultrasound was reported to be used daily to weekly, primarily for diagnostic purposes (Figure 2). All eight respondents agreed that pulmonary ultrasound during their fellowship meaningfully changed their approach to managing respiratory distress in critically ill patients.

## Discussion

Despite evidence of the growing importance of ultrasound in the management of critically ill patients, there remains no unified curriculum for POCUS training across critical care fellowship programs. Prior research highlights how structured ultrasound curricula can improve both confidence and proficiency among trainees, establishing a clear benefit to formalized instruction [6-7, 11,18-21]. However, much of this research has been confined to specific critical care subspecialties like surgical or pulmonary critical care, which may limit how broadly these findings apply. As the landscape of critical care becomes increasingly multidisciplinary, we anticipate a growing demand for POCUS curricula designed to accommodate learners from a variety of educational backgrounds. A recent expert consensus statement has provided comprehensive, evidence-based recommendations for longitudinal echocardiography training in critical care and could serve as a reference for future consensus guidelines that ultimately define a unified curriculum for critical care training programs [22].

In this study, we observed that fellows, regardless of their multidisciplinary residency training, were able to effectively apply LUS and accurately interpret images after longitudinal, integrated pulmonary ultrasound training. These findings suggest that integrating LUS into the fellowship curriculum through didactic sessions and hands-on training is feasible and likely an effective means to facilitate the acquisition of POCUS skills integral in the management of critically ill patients. Recent studies have explored blended learning models that combine online and in-person instruction to enhance ultrasound education for trainees [18-21]. Another study incorporated artificial intelligence into LUS education to assist inexperienced clinicians in acquiring high-quality ultrasound clips [23]. While our program emphasized intensive hands-on training, future curricula could consider blended approaches to optimize learning outcomes, especially when time or clinical availability is limited.

Previous studies, such as the one by Beaulieu et al. [24], report that providing 2.5 hours of theoretical education and two hours of hands-on training to junior emergency medicine residents had a significant impact on their ability to perform LUS. We obtained similar results, and fellows improved their knowledge and confidence in using LUS, with retention persisting up to one year. Fellows gained confidence in the integration of POCUS LUS with cardiac ultrasound for evaluating pulmonary pathologies, pleural effusion, and respiratory distress. However, fellows do not feel comfortable using diaphragm and LUS to assess weaning from a ventilator or to confirm ETT placement. Understandably, fellows have concerns about the use of diaphragm and LUS to predict weaning from the ventilator, as its accuracy varies depending on the patient subpopulation [15]. While ultrasound can confirm ETT placement, assessing ETT depth may still require a chest X-ray, although some literature supports ultrasound for this purpose [25].

Our study has several limitations. Given the descriptive aims and pilot nature of this study, we did not conduct formal hypothesis testing to assess the statistical significance of the educational intervention. Additionally, this study evaluated the feasibility of integrating the training model within a single training center. Aside from meta-analyses, we are not aware of any published studies evaluating the impact of ultrasound curricula across multiple institutions. Future studies may improve external validity and generalizability by incorporating multiple training institutions; doing so would likely prove critical to the development of a standardized ultrasound curriculum by the ACGME. 

An additional limitation is our small sample size, which limits robust evaluation of potential confounders impacting the internal validity of our study. For example, we did not have a sufficient sample size to assess the possible impact of previous training experience on pre- and post-training confidence. Additionally, there may be bias among survey respondents, as 42% had prior training in advanced ultrasound. As a result, the group may have been more inclined to integrate ultrasound into their daily practice. Finally, given that the focus of this study was to assess feasibility and impact on trainee confidence, we did not evaluate the impact of acquired pulmonary US skills on patient care. An important next step would be to assess the impact of this training intervention on patient outcomes. Future studies could benefit from more sophisticated learner assessments, such as Objective Structured Clinical Examinations (OSCE), and repeat assessments beyond one year. 

## Conclusions

Our study demonstrates the feasibility and effectiveness of integrating LUS into a multidisciplinary critical care fellowship curriculum. Fellows improved their knowledge, confidence, and efficiency in using pulmonary ultrasound, with sustained retention up to one year post-training. Future studies should assess the impact of POCUS training on patient outcomes and explore strategies to enhance proficiency in ventilator weaning and ETT assessment.

## Figures and Tables

**Figure 3:**
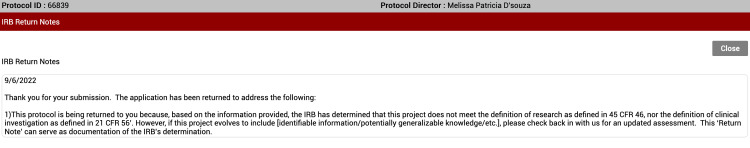
Proof of IRB exemption IRB: Institutional Review Board

## Appendices

Appendix A

Figure 3 showcases a snapshot of the IRB exemption.

Appendix B

The link to the 28-question pre-training survey to assess demographics, confidence, and knowledge of POCUS LUS: https://stanforduniversity.qualtrics.com/jfe/preview/previewId/a84b66e2-195a-456c-ac8e-5817a0ccc02e/SV_d4CtWh8wuZn5z26?Q_CHL=preview&Q_SurveyVersionID=current 

## References

[1] Moore CL, Copel JA (2011). Point-of-care ultrasonography. N Engl J Med.

[2] Van Schaik GW, Van Schaik KD, Murphy MC (2019). Point-of-care ultrasonography (POCUS) in a community emergency department: an analysis of decision making and cost savings associated with POCUS. J Ultrasound Med.

[3] Jones T, Leng P (2016). Clinical impact of point of care ultrasound (POCUS) consult service in a teaching hospital: effect on diagnoses and cost savings. Chest.

[4] Lentz B, Fong T, Rhyne R, Risko N (2021). A systematic review of the cost-effectiveness of ultrasound in emergency care settings. Ultrasound J.

[5] Schott Schott, Wetherbee Wetherbee, Khosla Khosla (2024). Current use, training, and barriers to point-of-care ultrasound use in ICUs in the Department of Veterans Affairs. Chest Crit Care.

[6] Killu K, Coba V, Mendez M (2014). Model point-of-care ultrasound curriculum in an intensive care unit fellowship program and its impact on patient management. Crit Care Res Pract.

[7] Lim S, Morris A, Hallman M (2012). Development of a multidisciplinary point-of-care ultrasound curriculum for intensivists. Am J Respir Crit Care Med.

[8] Melamed R, Sprenkle MD, Ulstad VK, Herzog CA, Leatherman JW (2009). Assessment of left ventricular function by intensivists using hand-held echocardiography. Chest.

[9] Ha YR, Toh HC (2016). Clinically integrated multi-organ point-of-care ultrasound for undifferentiated respiratory difficulty, chest pain, or shock: a critical analytic review. J Intensive Care.

[10] (2023). ACGME program requirements for graduate medical education in pulmonary disease and critical care medicine. https://www.acgme.org/globalassets/pfassets/programrequirements/2024-prs/156_pulmonarydiseasecriticalcaremedicine_2024.pdf.

[11] Jalilvand A, Bhatt A, Kopanczyk R, Wahl W (2024). Formal ultrasound curriculum for surgical critical care fellows leads to improvement in comfort and skills in the intensive care unit. Surgery.

[12] Rajamani A, Shetty K, Parmar J, Huang S, Ng J, Gunawan S, Gunawan G (2020). Longitudinal competence programs for basic point-of-care ultrasound in critical care: a systematic review. Chest.

[13] Volpicelli G, Elbarbary M, Blaivas M (2012). International evidence-based recommendations for point-of-care lung ultrasound. Intensive Care Med.

[14] Lichtenstein DA (2014). Lung ultrasound in the critically ill. Ann Intensive Care.

[15] Llamas-Álvarez AM, Tenza-Lozano EM, Latour-Pérez J (2017). Diaphragm and lung ultrasound to predict weaning outcome: systematic review and meta-analysis. Chest.

[16] Sethi AK, Salhotra R, Chandra M, Mohta M, Bhatt S, Kayina CA (2019). Confirmation of placement of endotracheal tube — a comparative observational pilot study of three ultrasound methods. J Anaesthesiol Clin Pharmacol.

[17] Gottlieb M, Holladay D, Burns KM, Nakitende D, Bailitz J (2020). Ultrasound for airway management: an evidence-based review for the emergency clinician. Am J Emerg Med.

[18] Milius C, Jepson L, Maclaskey D, Pazdernik V, Kondrashova T (2023). Development of hands-on skills in diagnostics of lung diseases using ultrasonography in undergraduate medical education. Mo Med.

[19] Stoehr F, Yang Y, Müller L (2023). A blended learning approach for teaching thoracic radiology to medical students: a proof-of-concept study. Front Med (Lausanne).

[20] Witte M, Ott M, Schilling T, Müller M, Schmid S, Krohn A (2023). Implementing an interprofessional point-of-care ultrasound protocol for dyspneic patients in an emergency department as a blended learning concept — feasibility of employing thoracic ultrasound in shortness of breath. Front Med (Lausanne).

[21] Sharma A, Kumar G, Nagpal R (2024). Efficacy of an online lung ultrasound module on skill acquisition by clinician: a new paradigm. Front Pediatr.

[22] Rajamani A, Galarza L, Sanfilippo F (2022). Criteria, processes, and determination of competence in basic critical care echocardiography training: a Delphi process consensus statement by the learning ultrasound in critical care (LUCC) initiative. Chest.

[23] Baloescu C, Bailitz J, Cheema B (2025). Artificial intelligence-guided lung ultrasound by nonexperts. JAMA Cardiol.

[24] Beaulieu Y, Laprise R, Drolet P (2015). Bedside ultrasound training using web-based e-learning and simulation early in the curriculum of residents. Crit Ultrasound J.

[25] Gottlieb M, Berzins D, Hartrich M (2022). Diagnostic accuracy of ultrasound to confirm endotracheal tube depth. Am J Emerg Med.

